# Safety and efficacy of unilateral focused ultrasound pallidotomy on motor complications in Parkinson’s disease (PD): a systematic review and meta-analysis

**DOI:** 10.1007/s10072-024-07617-2

**Published:** 2024-06-06

**Authors:** Abdallah Abbas, Malak A. Hassan, Rahma Sameh Shaheen, Amna Hussein, Mostafa Hossam El Din Moawad, Mostafa Meshref, Ahmed M. Raslan

**Affiliations:** 1 Faculty of Medicine, Al-Azhar University, Damietta, Egypt; 2 Faculty of Medicine, Alexandria University, Alexandria, Egypt; 3 Faculty of Medicine, Benha University, Benha, Egypt; 4 Department of Neurosurgery, University of Arizona College of Medicine, Phoenix, Arizona United States; 5 BSc Faculty of Pharmacy Clinical Department, Alexandria University, Alexandria, Egypt; 6 MSc Faculty of Medicine, Suez Canal University, Ismailia, Egypt; 7 Department of Neurology, Faculty of Medicine, Al-Azhar University, Cairo, Egypt; 8 Department of Neurological Surgery, Oregon Health & Science University, Portland, OR United States

**Keywords:** Focused ultrasound, Pallidotomy, Parkinson’s disease, Motor complications, Meta-analysis

## Abstract

To systematically review and conduct a meta-analysis to evaluate the safety and efficacy of the unilateral focused ultrasound (FUS) pallidotomy on motor complications in Parkinson’s disease (PD) patients. A comprehensive search strategy was implemented through August 15, 2023, and updated on February 13, 2024, across six databases, identifying studies relevant to unilateral focused ultrasound pallidotomy and PD. Eligibility criteria included observational studies, clinical trials, and case series reporting on the impact of the intervention on motor complications in PD patients. The screening and data extraction were done by two independent reviewers. Risk of bias assessment utilized appropriate tools for different study designs. Statistical analysis involved narrative synthesis and meta-analysis. Subgroup analyses and leave-one-out analyses were performed. Five studies were included in our study, involving 112 PD patients undergoing FUS pallidotomy. UPDRS-II analysis revealed a significant improvement from baseline (mean difference (MD): -3.205, 95% CI: -4.501, -1.909, *P* < 0.001). UPDRS-III overall change was significant (MD: -10.177, 95% CI: [-12.748, -7.606], *P* < 0.001). UPDRS-IV showed a significant change from baseline (MD: -5.069, 95% CI: [-5.915, -4.224], *P* < 0.001). UDysRS demonstrated a significant overall improvement (MD: -18.895, 95% CI: [-26.973, -10.818], *P* < 0.001). The effect of FUS pallidotomy on motor complications in PD patients was effective, with a significant decrease in the UPDRS and UDysRS, reflecting improvement. The incidence of adverse events (headaches, pin-site pain, difficulty walking, and sonication-related head pain) of the FUS pallidotomy was not statistically significant, indicating its safety.

**Supplementary Information:**

The online version contains supplementary material available at 10.1007/s10072-024-07617-2.

## Introduction

Parkinson’s disease (PD) is a neurodegenerative disorder that affects the central nervous system and is characterized by a progressive loss of dopaminergic neurons in the substantia nigra of the brain [1]. The primary features of PD include tremors, rigidity, bradykinesia, and postural instability, in addition to a host of other associated symptoms [1]. Parkinson’s disease is a growing condition, especially in the aging population, with a prevalence of 1% of individuals over 60 and up to 4% in higher age groups [2]. Parkinson’s disease has a significant impact on individuals and society, leading to a decline in quality of life, increased healthcare costs, and caregiver burden [3].

The existing treatment modalities for PD include medication, physical therapy, and surgical interventions, including deep brain stimulation (DBS) [4, 5]. Levodopa is the most commonly used medication for PD, which helps to increase dopamine levels in the brain [4]. However, the long-term use of levodopa can lead to motor complications such as dyskinesia and motor fluctuations [6]. Two main approaches for treating PD through surgery are ablation and modulation. Ablation entails performing thalamotomy for PD primarily characterized by tremor-predominant Parkinson’s disease (TDPD) and pallidotomy for conventional PD [7]. Modulation is achieved currently using DBS, which is a surgical intervention that involves implanting electrodes in the brain to stimulate specific areas, which can help alleviate motor symptoms [8]. Thalamotomy and pallidotomy are traditionally carried out through radiofrequency (RF) lesioning, which involves invasive access through a small hole in the cranium and penetration with an RF probe [9]. Procedures that involve intrusion, such as RF thalamotomy and DBS, carry the potential for complications like infection, bleeding, and malfunction of hardware in the case of DBS [10].

In order to create lesions in the brain, high-frequency focused ultrasound (FUS) offers a non-invasive approach. In the United States, an FDA-approved system is available for performing thalamotomy for essential tremor (ET), thalamotomy for TRPD, and unilateral pallidotomy for PD [11]. Focused ultrasound pallidotomy is an innovative treatment approach for PD that involves using high-intensity ultrasound waves to create a lesion in the globus pallidus, a region of the brain that is overactive in PD [12]. The lesion helps to reduce the abnormal activity in the globus pallidus, which can alleviate motor symptoms [12]. Focused ultrasound pallidotomy is a non-invasive procedure and potential alternative treatment approach that does not require surgery, and it has shown promising results in clinical trials [13, 14].

At this time, focused ultrasound is only approved for unilateral pallidotomy of medication-refractory Parkinson's patients with moderate to severe tremors or dyskinesia [15]. However, studies are being organized to assess the possibility of treating patients bilaterally [15]. A single case report showed that bilateral focused ultrasound pallidotomy improved bradykinesia and rigidity with a stable gait and good postural reflexes in a PD patient with facial dyskinesia [12].

There is currently a plethora of literature and long-term data from a randomized controlled trial for FUS treatment in ET and, to a lesser extent, for TRPD [13, 16, 17]. However, FUS pallidotomy remains a relatively innovative method with emerging evidence of efficacy, and as such, there is a need for a systematic review and meta-analysis of unilateral focused ultrasound pallidotomy for motor complications in PD. A systematic review and meta-analysis can help to evaluate the effectiveness and safety of unilateral focused ultrasound pallidotomy and provide insights into its potential as a treatment option for PD.

## Methods

All steps carried out in this study were executed with utmost adherence to the Cochrane Handbook of Systematic Reviews of Interventions [18]. Furthermore, the PRISMA statement recommendations were adhered to while presenting the findings of this systematic review and meta-analysis, as stated in reference [19]. The protocol was registered in PROSPERO (CRD42023474216).

### Search strategy and screening

Six databases (PubMed, Scopus, Web of Science, Embase, Ovid, and Medline) were searched through August 15, 2023, and updated on February 13, 2024, without using any filters, using the following query: ((“focused ultrasound pallidotomy” OR “HIFU pallidotomy” OR “MRgFUS pallidotomy” OR “MRgFUS ablation” OR “globus pallidus focused ultrasound ablation”) AND (“parkinso*” OR “PD” OR “hypokinetic rigid syndrome” OR “bradykinesia-rigidity syndrome”)). The search process was done by two authors (M.A.H. and R.S.S.), who referred any conflicts to a third one (A.A.).

Eligibility criteria included all the observational studies, clinical trials, and case series that reported the effect of unilateral focused ultrasound pallidotomy on motor complications in patients with PD. We excluded all case reports and studies from which data could not be reliably extracted.

Two independent reviewers (M.A.H. and R.S.S.) conducted the screening using Rayyan software [20]. The screening involved two steps, which were (a) title and abstract screening to ensure they were in line with the inclusion criteria and (b) full-text screening to determine eligibility for quantitative analysis. If there were any conflicts, they were resolved through discussion and consensus, with input from a third author (A. A.) if necessary.

### Data extraction

Two authors extracted the relevant data using Microsoft Excel 2021 [21]. The extracted data included the following: (a) characters of study design; (b) characteristics of study patients, including (age, sex, years since the diagnosis with PD, and daily levodopa equivalents); (c) risk of bias domains; and (d) study outcomes, including efficacy outcomes (UPDRS and UDysRS) and safety outcomes (headache, pin-site pain, dysarthria, difficulty walking or imbalance, and sonication-related head pain). If any inconsistencies in the extraction of data were found, they were resolved through discussion or consultation with a third author, if necessary.

### Risk of bias assessment

A bias assessment was conducted by two authors working independently, and in case of any disagreement, a third author was consulted. Depending on the study design, different tools were used to conduct the assessment: Revised Cochrane risk-of-bias tool (RoB.2) for RCTs, Methodological Index for Non-Randomized Studies (MINORS) for single-arm non-randomized clinical trials, Newcastle-Ottawa scale (NOS) for observational studies, and NIH tool for case series.

The RoB.2 tool [22] is divided into five domains, each with a set of questions that can be answered with "yes," "no," "possibly yes," "possibly no," or "no information." The results are combined through a diagram to give one of three levels of bias: low risk, some concern, or high risk of bias. If all five domains have a low risk of bias, then the study has a low risk of bias. If at least one domain has some concerns, then the study is reported to have some concerns about bias. If at least one domain has a high risk of bias or multiple domains have some concerns, then the study is reported to have a high risk of bias.

The MINORS [23] tool is comprised of eight domains for non-comparative studies. If a domain is not reported, it receives a score of 0. If it is inadequately reported, it receives a score of 1. If it is adequately reported, it receives a score of 2. In non-comparative studies, the maximum score is 16. Based on the cut-off points, if the score is ≤ 8, the study quality can be considered poor. If it is 9–14, it can be considered moderate, and if it is 15–16, it can be considered good.

The NOS tool [24] evaluates each observational study based on nine items divided into three groups: the selection of study participants, the comparability of groups, and the ascertainment of either exposure or outcome of interest. Each item is answered with either 'yes' or 'no'. Studies with scores of 7–9 are considered good quality, while those with scores of 5–6 are of fair quality. Scores of 1–4 are considered poor quality.

Lastly, the quality of a study based on the NIH tool [25] is evaluated using nine items. If the answer is 'yes,' it receives a score of 1; if it is not, it receives a score of 0. If it cannot be determined (CD), it receives a score of CD; if it is not applicable, it receives a score of NA; and if it is not reported, it receives a score of NR. Good quality is assigned to studies with scores of 9–8, fair quality is assigned to studies with scores of 7–6, and poor quality is assigned to studies with scores of 1–5.

### Statistical analysis

To summarize the findings of the included studies, a narrative synthesis was conducted, which provided details regarding study characteristics, population, intervention, and outcomes.

The Meta Converter tool [26] was utilized to convert between different statistical variables, such as mean and standard deviation, and to calculate the change from baseline, while OpenMetaAnalyst software [27] was used to conduct the meta-analysis. Continuous data was pooled as the mean difference (MD) between the post-treatment and baseline data with a 95% confidence interval (CI). On the other hand, the qualitative data was pooled as the overall event rate.

Heterogeneity was assessed using the Chi-square test, and its extent was measured using the I-square test. If there was insignificant heterogeneity, we used the fixed-effects model, while significant heterogeneity (Chi-square *p* < 0.1) led us to conduct the analysis under the random effects model [18, 28]. The mean difference in the change from baseline in the focused ultrasound pallidotomy group was the effect size used.

To precisely evaluate the effect of the unilateral focused ultrasound pallidotomy on motor complications, a subgroup analysis was conducted by follow-up duration. OpenMetaAnalyst software [27] was used to perform a leave-one-out analysis in case of significant heterogeneity to evaluate the robustness of the findings by excluding studies with a high risk of bias. Due to the number of included studies being less than ten [29], publication bias could not be assessed.

## Results

### Search strategy and screening

**Fig. 1:**
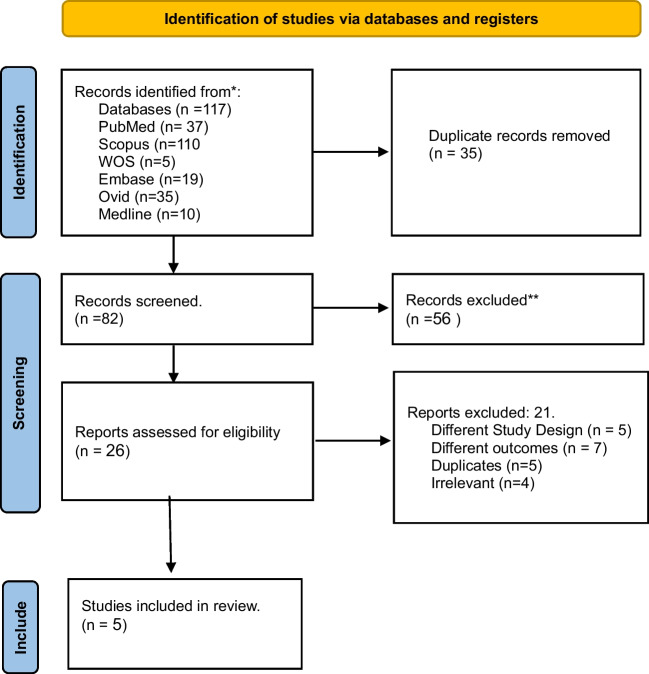
Shows the PRISMA flow diagram of the search and screening for the included studies

After conducting our initial search, we found 117 studies. Once we had resolved any duplicates, we were left with a total of 82 studies. We then proceeded to meticulously screen the abstracts of these studies using our predetermined inclusion and exclusion criteria. After thoroughly examining the full text and references of 26 studies, we determined that one study [30] was suitable for inclusion in our systematic review, while four studies [13, 14, 16, 17] were deemed suitable for inclusion in our meta-analysis (Fig. 1).

### Baseline characteristics

The five studies included a total of 112 patients with PD who were selected to undergo FUS pallidotomy. Of the 112 patients, 69 were males. Though the studies were from various countries, it is worth noting that 99 participants were in the United States (Table 1).

**Table 1:** Shows the summary and baseline characteristics of the included studies

Study ID	Study design	Country	Sample size	Age, mean (SD)	Sex (males), N (Total)	Follow-up duration	Daily L-dopa equivalents (mg), mean (SD) or [range]	Main findings
Jung et al. 2019 [14]	Prospective, nonrandomized, single-arm clinical trial	Korea	10	59.8	4 (10)	12 months	1156 [738-1750]	MRgFUS improved motor outcomes and quality of life. MRgFUS was found to be a safe procedure.
Sammartino et. al 2022 [30]	Clinical trial	United States	11	62.8 (9.3)	9 (11)	NA	NA	MRgFUS improved clinical outcomes. The authors used clinical data and radiological methods to determine globus pallidus motor subregions to optimize targeting accuracy.
Eisenberg et al. 2021 [13]	Open-label clinical trial	United States	20	56.4 (11.3)	13 (20)	12 months	1039.4 (601.3)	The primary efficacy outcome measure, the total UDysRS score, showed a significant 59% improvement from its baseline at three months (*p* < 0.0001, using a paired t-test).
Ito et al. 2020 [17]	Single-center, prospective, and open-labeled study	Japan	3	69 (9.5)	0 (3)	24 months	1086.2 (284.9)	Two patients showed improvements in motor fluctuations, but one experienced worsening of motor function in both the off-medication state and when taking levodopa, which led to exacerbated dyskinesia. The other patient had a two-year improvement in levodopa-induced dyskinesia but limited improvement in motor function, which was eventually exacerbated. Both patients remained free of serious and delayed complications.
Krishna et al. 2023 [16]	Multicenter, prospective, double-blind, randomized, sham-controlled trial	United States	68	64.2 (9.6)	43 (68)	12 months	1051.6 (473.8)	After three months, patients who underwent unilateral pallidal ultrasound ablation experienced better motor function or reduced dyskinesia despite some adverse effects.

*SD* Standard Deviation, *N* Number of patients, *mg* milligrams, *NA* Not Answered, *MRgFUS* magnetic resonance imaging-guided focused ultrasound

### Risk of bias assessment

One study [16] was assessed by the RoB.2 tool with an overall “some concerns” risk of bias (Supplementary Fig. 1). Two studies [13, 14] were assessed using the MINORS tool, with moderate quality in both (Supplementary Table 1). One study [30] was assessed via the NOS tool with good quality (Supplementary Table 2). One study [17] was assessed using the NIH tool with an overall fair quality (Supplementary Table 3).

### Statistical analysis

#### UPDRS-II

**Fig. 2:**
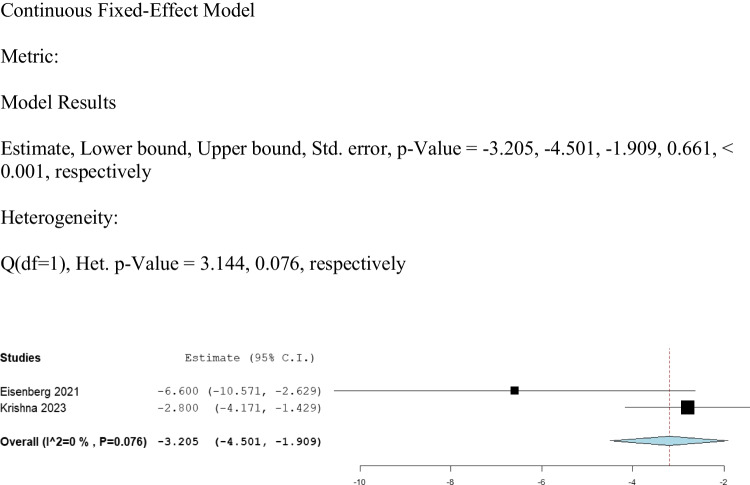
Shows the analysis of UPDRS-II

We analyzed the data provided by Eisenberg 2021 [13] and Krishna 2023 [16], which involved 88 patients on UPDRS-II, as shown in Fig. 2. The results showed a statistically significant improvement (mean difference (MD): -3.205, 95% CI: [-4.501, -1.909], *P* < 0.001) in comparison to the baseline data (Fig. 2). The data was homogeneous (I^2 = 0%, Het. P = 0.076).

#### UPDRS-III

**Fig. 3:**
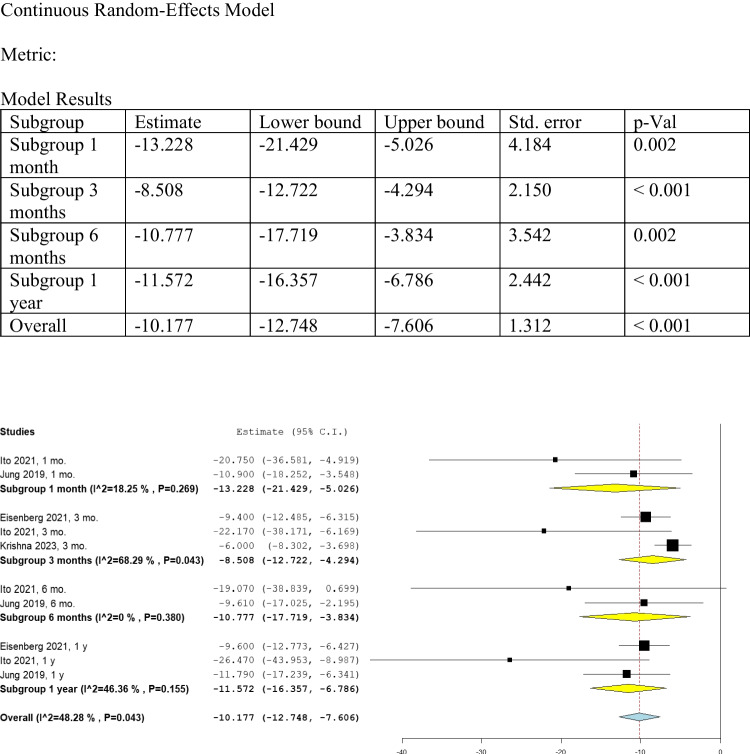
Shows the analysis of UPDRS-III

We conducted a subgroup analysis to evaluate the change in UPDRS-III scores from baseline at one month, three months, six months, and one year (Fig. 3). With a total of 101 patients, the overall analysis of these subgroups indicated a statistically significant improvement of UPDRS-III compared with baseline data (MD: -10.177, 95% CI: [-12.748, -7.606], *P* < 0.001) (Fig. 3).

##### UPDRS-III at one month

Ito 2021 [17] and Jung 2019 [14], with a total of 13 patients, provided data on UPDRS-III scores after one month of the pallidotomy procedure. There was a statistically significant improvement after FUS pallidotomy (MD: -13.228, 95% CI: [-21.429, -5.026], P = 0.002) compared with baseline data. The data was homogenous (I^2 = 18.25%, Het. *P* = 0.269) (Fig. 3).

##### UPDRS-III at three months

Three studies, Eisenberg 2021 [13], Ito 2021 [17], and Krishna 2023 [16], with a total of 91 patients, provided information on UPDRS-III scores three months after the pallidotomy procedure. The data showed a statistically significant improvement of (MD: -8.508, 95% CI: [-12.722, -4.294], *P* < 0.001) when compared to the baseline data (Fig. 3). However, the data was heterogeneous at three months (I^2 = 68.29%, Het. *P* = 0.043). Therefore, we conducted a leave-one-out analysis for UPDRS-III at three months, which showed that the studies from Eisenberg 2021 [13] and Krishna 2023 [16] were potential sources of heterogeneity. This was because their effect sizes were significantly different from the overall effect size (Supplementary Fig. 2).

##### UPDRS-III at six months

Ito 2021 [17] and Jung 2019 [14], with a total of 13 patients, provided data on UPDRS-III scores after six months of the pallidotomy procedure. The change from baseline was found to be statistically significant (MD: -10.777, 95% CI: [-17.719, -3.834], *P* = 0.002) (Fig. 3). The data was homogenous (I^2 = 0%, Het. *P* = 0.380).

##### UPDRS-III at one year

Eisenberg 2021 [13], Ito 2021 [17], and Jung 2019 [14], with a total of 33 patients, provided data on UPDRS-III scores after one year from the pallidotomy procedure. The change from baseline was found to be statistically significant (MD: -11.572, 95% CI: [-16.357, -6.786], *P* < 0.001) (Fig. 3), indicating a significant improvement after treatment with FUS pallidotomy compared with baseline data. No significant heterogeneity was found at one year (I^2 = 46.36%, Het. *P* = 0.155).

#### UPDRS-IV

**Fig. 4:**
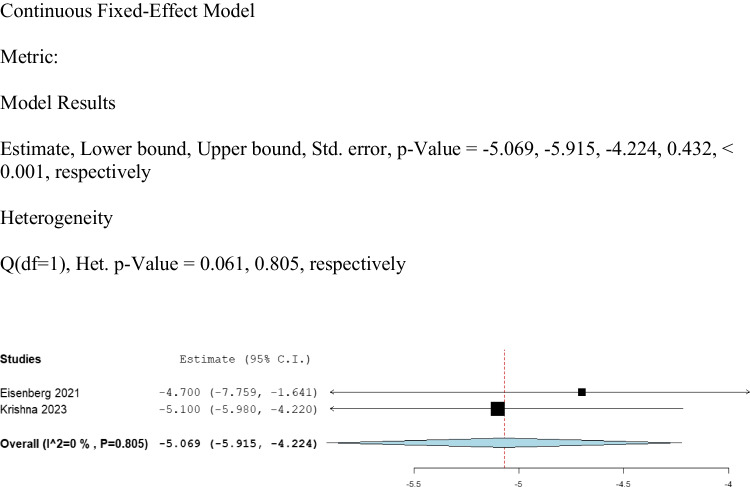
Shows the analysis of UPDRS- IV

We analyzed the change from baseline for UPDRS-IV (Fig. 4). Eisenberg 2021 [13] and Krishna 2023 [16], which involved 88 patients, provided data on UPDRS-IV scores. The change from baseline was found to be statistically significant at (MD: -5.069, 95% CI: [-5.915, -4.224], *P* < 0.001). No significant heterogeneity was found (I^2 = 0%, Het. *P* = 0.805).

#### UDysRS

**Fig. 5:**
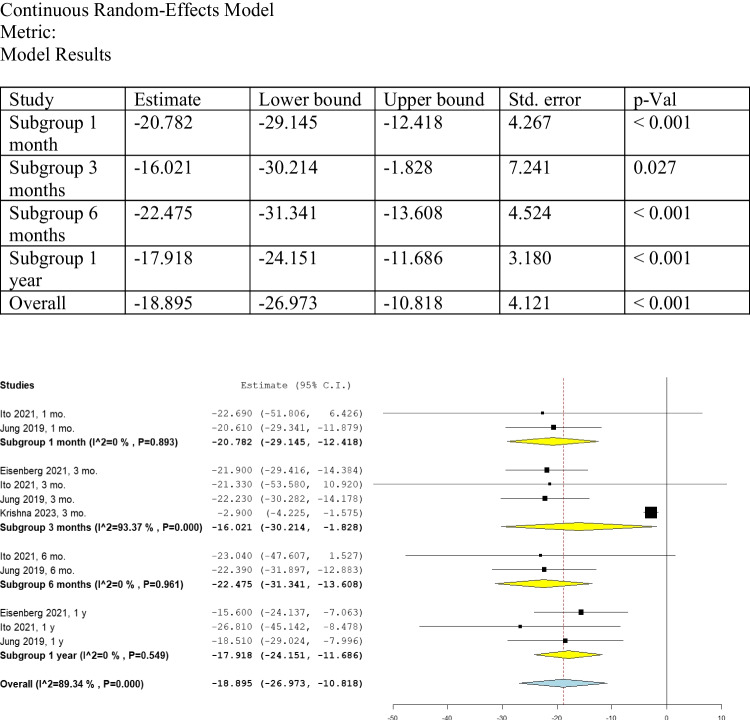
Shows the analysis of UDysRS

We analyzed the change from baseline for UDysRS at one month, three months, six months, and one year. The overall analysis of these subgroups, which involved 101 patients, was found to be statistically significant (MD: -18.895, 95% CI: [-26.973, -10.818], *P* < 0.001) (Fig. 5), indicating that the treatment with FUS pallidotomy significantly improved the UDysRS compared to baseline data.

##### UDysRS at one month

Ito 2021 [17] and Jung 2019 [14], which involved 13 patients, provided data on UDysRS scores one month after the pallidotomy procedure. The change from baseline was found to be statistically significant (MD: -20.782, 95% CI: [-29.145, -12.418], *P* < 0.001) (Fig. 5). No significant heterogeneity was found at one month (I^2 = 0%, Het. *P* = 0.893).

##### UDysRS at three months

Eisenberg 2021, Ito 2021, Jung 2019, and Krishna 2023 [13, 14, 16, 17], with a total of 33 patients, provided data on UDysRS scores after three months of the pallidotomy procedure. The change from baseline was found to be statistically significant (MD: -16.021, 95% CI: [-30.214, -1.828], *P* = 0.027) (Fig. 5). Heterogeneity was found at three months (I^2 = 93.37%, Het. *P* = 0.000). Thus, we conducted a leave-one-out analysis for UDysRS after three months and found that the source of heterogeneity was Krishna 2023 [16] (Supplementary Fig. 3).

##### UDysRS at six months

Ito 2021 [17] and Jung 2019 [14], with a total of 13 patients, provided data on UDysRS scores after six months of the pallidotomy procedure. The change from baseline was found to be statistically significant (MD: -22.475, 95% CI: [-31.341, -13.608], *P* < 0.001) (Fig. 5). No significant heterogeneity was found at six months (I^2 = 0%, Het. *P* = 0.961).

##### UDysRS at one year

Eisenberg 2021, Ito 2021, and Jung 2019 [13, 14, 17], with a total of 33 patients, provided data on UDysRS scores after one year of the pallidotomy procedure. The change from baseline was found to be statistically significant (MD: -17.918, 95% CI: [-24.151, -11.686], *P* < 0.001) (Fig. 5). No significant heterogeneity was found at one month (I^2 = 0%, Het. *P* = 0.549).

### Safety outcomes

Using data from the included studies, we analyzed the incidence rate of various adverse effects that were common in at least two of the studies. In addition, adverse events were summarized in Table 2, including the number of studies reporting each event, the total number of events, and the percentage of occurrence.

**Table 2:** Summary of adverse events

Adverse event	Studies reported	Incidence rate in the FUS pallidotomy group (Event/Total, (%))
Headaches	3	16/98, (16%)
Pin-site pain or complications	2	19/78, (24%)
Dysarthria	3	7/98, (7%)
Difficulty walking or imbalance	2	3/88, (3%)
Sonication-related head pain	2	8/88, (9%)

#### The incidence rate of headaches

In three studies, Jung 2019, Eisenberg 2021, and Krishna 2023 [13, 14, 16], a total of 98 participants reported headaches as an adverse event. Headaches occurred in 16 of the 98 participants (Supplementary Fig. 4). The occurrence of headaches was not statistically significant (Event Rate (ER): 0.382, 95% CI: [-0.198, 0.962], *P* = 0.196) (Supplementary Fig. 4), indicating that the FUS pallidotomy was safe on the headache level since the null hypothesis was zero response. The data was heterogeneous (I^2 = 98.9%, Het. *P* < 0.001). Thus, we conducted a leave-one-out analysis and found that the source of heterogeneity was Jung 2019 (Supplementary Fig. 5).

#### The incidence rate of pin-site pain or complications

In two studies, Jung 2019 and Krishna 2023 [14, 16], a total of 78 participants reported pin-site pain or complications as an adverse event. Pin-site pain or complications occurred in 19 of the 78 participants (Supplementary Fig. 6). This incidence was not statistically significant (ER: 0.542, 95% CI: [-0.264, 1.348], *P* = 0.187) (Supplementary Fig. 6), so the FUS pallidotomy was safe when it comes to pain-site pain.

#### The incidence rate of dysarthria

In three studies, Jung 2019, Eisenberg 2021, and Krishna 2023 [13, 14, 16], a total of 98 participants reported dysarthria as an adverse event. Dysarthria occurred in seven of the 98 participants (Supplementary Fig. 7). The occurrence of dysarthria was statistically significant (ER: 0.041, 95% CI: [0.002, 0.079], *P* = 0.038) (Supplementary Fig. 7). Hence, the FUS pallidotomy was not safe regarding dysarthria. The data was homogenous (I^2 = 0%, Het. *P* = 0.145).

#### The incidence rate of difficulty walking or imbalance

Two studies, Eisenberg 2021 and Krishna 2023, with a total of 88 participants, reported difficulty walking or imbalance as an adverse event. Difficulty walking or imbalance occurred in three of the 88 participants (Supplementary Fig. 8). The FUS pallidotomy was safe with an insignificant incidence of difficulty walking (ER: 0.033, 95% CI: [-0.005, 0.070], *P* = 0.085).

#### The incidence rate of sonication-related head pain

Two studies, Eisenberg 2021 and Krishna 2023 [13, 16], with a total of 88 participants, reported sonication-related head pain as an adverse event. Sonication-related head pain occurred in eight of the 88 participants (Supplementary Fig. 9). Our analysis did not find this statistically significant (ER: 0.166, 95% CI: [-0.161, 0.493], *P* = 0.321), revealing that the FUS pallidotomy was safe regarding the sonication-related head pain.

## Discussion

As a technique for CNS ablation, focused ultrasound offers appealing benefits such as non-invasiveness and real-time feedback. These two characteristics address the traditional challenges of other ablative methods, such as radiosurgery's lack of real-time feedback and radiofrequency's invasiveness [31, 32]. DBS is presently the most common surgical treatment for PD, thanks to its ability to be both reversible and scalable [31, 32].

FUS is becoming more popular because it can treat patients who are not suitable for surgery, and it is relatively safe compared to other invasive options despite its irreversible nature [31, 32]. Evidence suggests that the use of focused ultrasound is both safe and effective for treating ET and TDPD [32]. FUS-based CNS ablation techniques that are approved by the FDA include thalamotomy for ET, thalamotomy for TDRD, and pallidotomy for PD [33]. Moreover, MR-guided high-intensity focused ultrasound (HIFU) subthalamotomy is a promising new treatment for PD and other forms of parkinsonism [34]. A recent RCT has demonstrated the effectiveness and safety of this minimally invasive procedure [35]. This technique targets the subthalamic nucleus, offering a promising alternative for patients with conventional PD [34]. However, it's worth noting that while this approach shows potential, further research is needed to establish its efficacy and safety profile conclusively.

The symptoms and targets currently assessed for PD include focusing solely on PD. These targets consist of 1) thalamotomy, which targets Parkinsonian tremor in the thalamus. 2) pallidotomy or subthalamic nucleus targeting Parkinsonian dyskinesia in the globus pallidus, and 3) the pallidothalamic tract targeting Parkinsonian tremor, akinesia, or dyskinesia [15].

Focused ultrasound provides a one-time solution without the need for follow-up procedures or visits for battery replacement, wire repair, or simulator adjustments, which is different from deep brain stimulation. Additionally, it does not harm healthy tissue or carry the risk of infections that come with implanting a foreign object [36].

In comparison with baseline data, our findings revealed that the FUS pallidotomy significantly improved UPDRS-II, UPDRS-III, and UPDRS-IV, and total UDysRS across all the durations that were analyzed (1 month, three months, six months, and one year), indicating a valuable improvement to the motor symptoms in patients with PD.

These results were consistent with most of the literature. Eisenberg et al. [13] found that MRgFUS pallidotomy was a safe and effective treatment option for patients with PD who had asymmetrical symptoms and a fluctuating response to medication, including dyskinesia. The study showed significant improvement in the two scores, UPDRS and UDysRS, including a 52.7% improvement in total UDysRS scores and a 30.2% reduction in UPDRS scores at six months. The study also demonstrated a high level of safety and tolerance, with minimal neurological adverse events and no intracranial bleeding or infection. However, this study had some limitations that should be considered; the small sample size, which was only 27 patients, was included in the analysis. Additionally, the study lacked a control group, which limited the ability to compare the outcomes of MRgFUS pallidotomy to other treatment options.

Krishna et al. [16] found that the unilateral pallidal ultrasound ablation resulted in a higher percentage of patients who had improved motor function or reduced dyskinesia than a sham procedure over a period of 3 months but was associated with adverse events. However, there were some limitations to this study that should be considered. First, there were adverse neurological effects associated with the unilateral pallidal ultrasound ablation. Additionally, there were also missing data for primary and secondary outcomes, which were addressed through sensitivity analysis.

Ito et al. [17] found that MRgFUS unilateral pallidotomy in patients with PD resulted in improvements in motor function and dyskinesia. The improvements in UPDRS part III scores and UDysRS scores were observed at 1, 3, 6, 12, and 24 months after pallidotomy. The study also reported variations in the post-treatment course among the patients, with some experiencing exacerbations of motor fluctuations and dyskinesia. However, overall, the procedure was considered safe and effective for treating medication-refractory motor fluctuations and dyskinesia in PD patients. However, this study included only three patients, which might have affected the results.

Jung et al. [14] have found that MRgFUS pallidotomy can be used to control cardinal motor symptoms in patients with PD. However, further investigation and continuous follow-up are necessary to clarify the indications for MRgFUS pallidotomy, as well as its safety and long-term effectiveness. However, this study showed some limitations, including the small number of patients, lack of randomization, and absence of a control group. The study was underpowered, which may have contributed to the lack of statistical significance.

When analyzing the safety outcomes in our study, we found that the FUS pallidotomy was safe with an insignificant occurrence of headaches, pin-site pain, difficulty walking, and sonication-related head pain, but the occurrence was not safe on the dysarthria level.

A study was conducted by Fishman et al. [37] to analyze the safety profile of MRgFUS thalamotomy for ET. The findings of the study showed that the majority of adverse events associated with MRgFUS thalamotomy were rated as mild or moderate in severity.

One strength of our study is the subgroup analysis conducted for each duration, from the first month to one year, to provide guidance on the effect of FUS pallidotomy on motor complications over time. We observed that the overall mean difference or the effect of FUS pallidotomy on the UPDRS and UDysRS decreased with time, but the difference remained statistically significant. Therefore, we recommend that future research increase the follow-up duration to determine the long-term effect of FUS pallidotomy.

A limitation of our study is the inclusion of different study designs and a limited number of studies, which may affect the findings. However, we tried to handle this by conducting a subgroup analysis and leave-one-out analysis in case of significant heterogeneity to detect the source of heterogeneity and also using a random effects model with significant heterogeneity. Another limitation is our inability to compare FUS pallidotomy with a sham or control group. As a result, we conducted a single-arm meta-analysis by using the mean difference between the post-treatment FUS pallidotomy and baseline data.

For future research, it is recommended to conduct studies with larger sample sizes, strict sampling methods, a control group, and double-blinding to limit the bias. Secondly, we recommend increasing the follow-up duration to more than one year to determine the long-term effect of FUS pallidotomy.

## Conclusion

According to our study, FUS pallidotomy can be a transformative treatment for patients who are not suitable for surgery or have not responded to medications. The treatment significantly improves motor complications in PD patients, leading to a decrease in UPDRS and UDysRS scores, which reflect the improvement. Additionally, the treatment has a low incidence of adverse events, such as headaches, pin-site pain, difficulty walking, and sonication-related head pain, which are not statistically significant and indicate the safety of FUS pallidotomy. However, further studies are necessary to confirm these discoveries.

## Data Availability

Data were publicly available.

## Supplemenrray information

ESM 1 (DOCX 479 kb)

## Abbreviations

*PD*: Parkinson’s Disease
*DBS*: Deep Brain Stimulation
*RF*: Radiofrequency
*FUS*: Focused Ultrasound
FDA: U.S. Food and Drug Administration
*ET*: Essential Tremor
*TRPD*: Tremor-Predominant Parkinson’s Disease
*HIFU*: High-Intensity Focused Ultrasound
*MRgFUS*: Magnetic Resonance-guided Focused Ultrasound
*UPDRS*: Unified Parkinson’s Disease Rating Scale
*UDysRS*: Unified Dyskinesia Rating Scale
*PRISMA*: Preferred Reporting Items for Systematic Reviews and Meta-Analyses
*RCT*: Randomized Controlled Trial
*RoB.2*: Revised Cochrane risk-of-bias tool
*MINORS*: Methodological Index for Non-Randomized Studies
*NOS*: Newcastle-Ottawa Scale
*NIH*: National Institutes of Health
*CNS*: Central Nervous System
*CI*: Confidence Interval
