# Supplementary material for: Safety and efficacy of unilateral focused ultrasound pallidotomy on motor complications in Parkinson’s disease (PD): a systematic review and meta-analysis
Source: Neurol Sci. 2024 Jun 6;45(10):4687–98. doi: 10.1007/s10072-024-07617-2 (PMC11422448; doi:10.1007/s10072-024-07617-2)
Supplement: ESM 1 — (DOCX 479 kb) [file 10072_2024_7617_MOESM1_ESM.docx]

**Supplementary Fig. 1. Shows the risk of bias assessment of the RCTs by the RoB.2 tool**


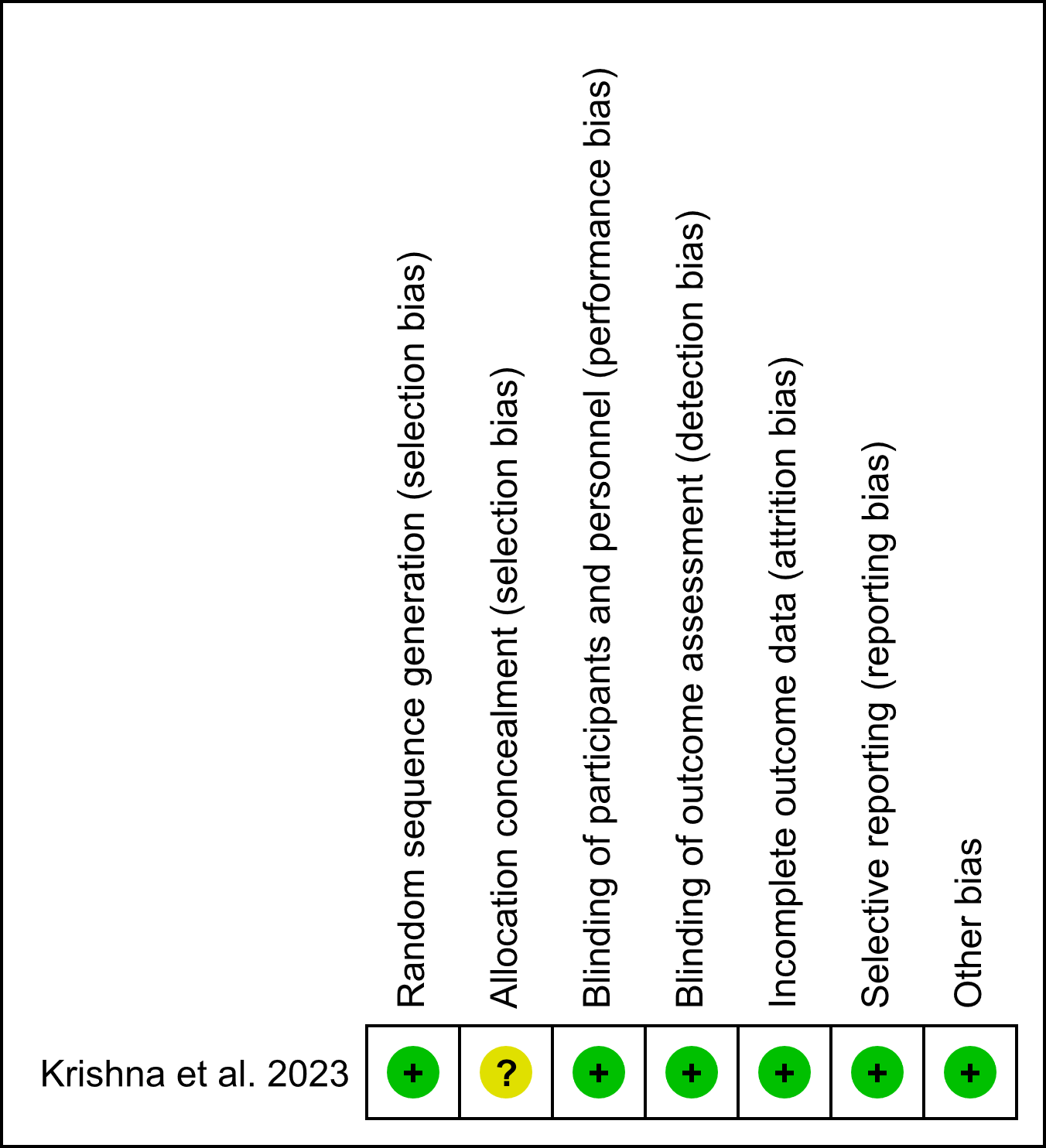


**Supplementary Fig. 2. Shows the leave-one-out analysis of UPDRS-III at 3 months**


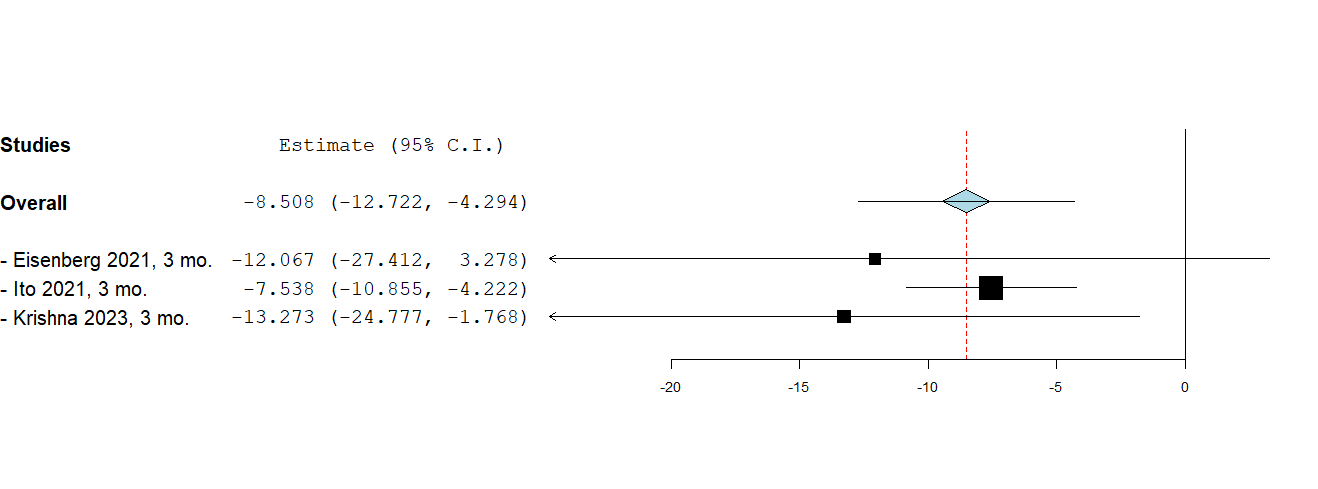


**Supplementary Fig. 3. Shows the leave-one-out analysis of UDysRS at 3 months**


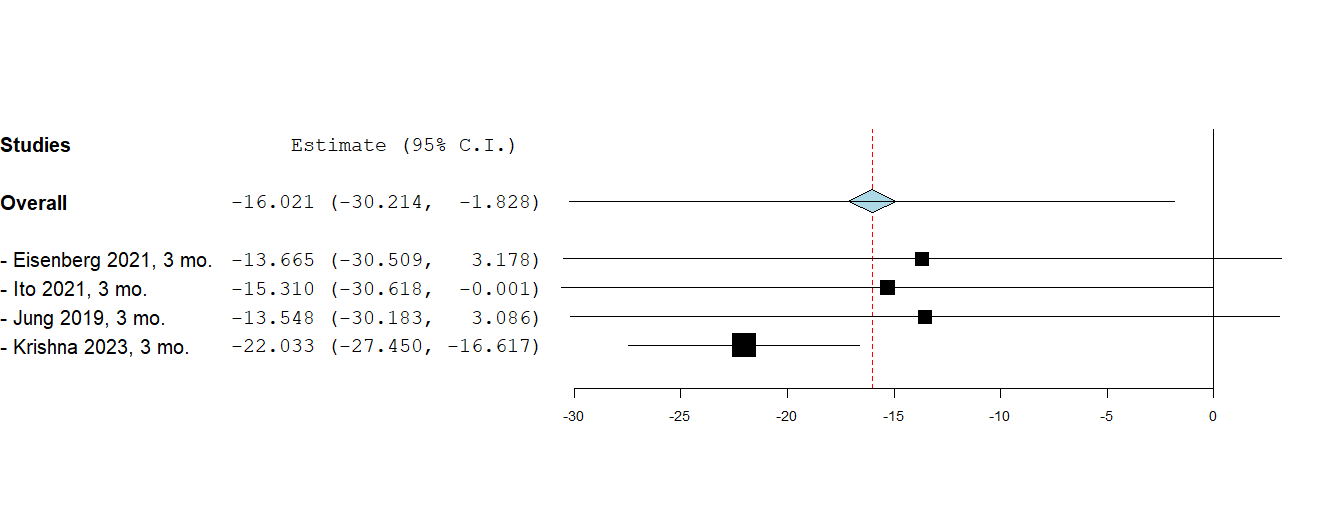


**Supplementary Fig. 4. Shows the overall incidence rate of headaches**

**Supplementary Fig. 5. Shows the leave-one-out analysis for the overall incidence rate of headaches**


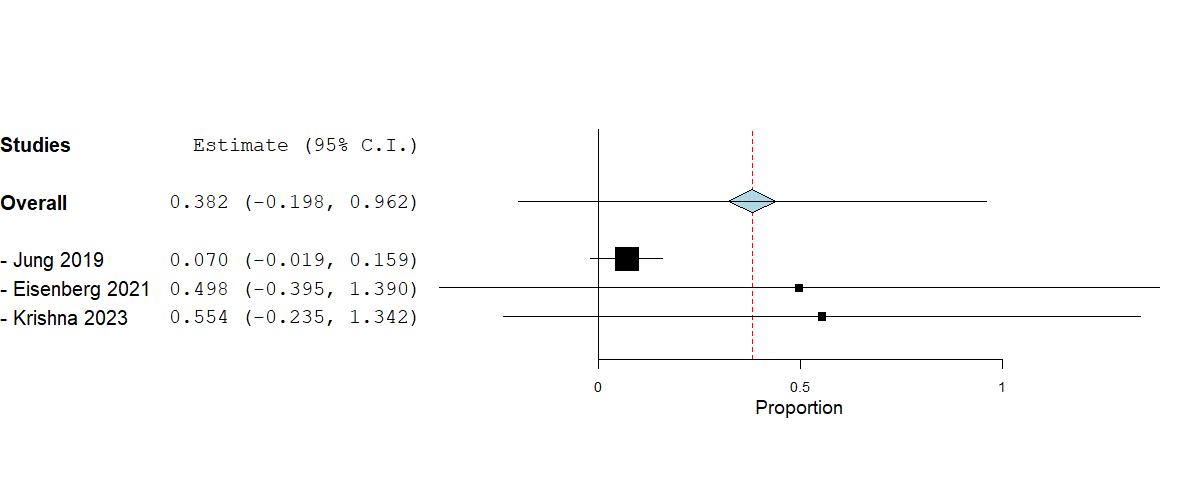


**Supplementary Fig. 6. Shows the overall incidence rate of pin-site pain or complications**

**Supplementary Fig. 7. Shows the overall incidence rate of dysarthria**

**Supplementary Fig. 8. Shows the overall incidence rate of difficulty walking or imbalance**

**Supplementary Fig. 9. Shows the overall incidence rate of sonication-related head pain**

**Supplementary Table 1. Shows the risk of bias assessment of single-arm clinical trials using the MINORS tool**

| Study ID | Eisenberg et al. 2021 | Jung et al. 2019 |
| --- | --- | --- |
| A clearly stated aim | 2 | 2 |
| Inclusion of consecutive  patients | 2 | 2 |
| Prospective collection of data | 0 | 1 |
| The endpoint appropriate to the study's aim | 2 | 2 |
| Unbiased evaluation of  endpoints | 0 | 0 |
| Follow-up period appropriate  to the major endpoint | 2 | 2 |
| Loss to follow up not  exceeding 5% | 2 | 2 |
| Prospective calculation of the study size | 2 | 0 |
| Total Score | 12 | 11 |

**Supplementary Table 2. Shows the risk of bias assessment of observational studies using the Newcastle-Ottawa scale tool**

| Study ID | Selection | | | | Comparability | Outcome | | | Score |
| --- | --- | --- | --- | --- | --- | --- | --- | --- | --- |
|  | exposed | non-exposed | Ascertainment of exposure | start without outcome present | Comparability | Outcome assessment | Follow up length | Adequacy of outcome |  |
| Sammartino et. al 2022 | - | * | * | * | ** | * | - | * | 7 |

**Supplementary Table 3. Shows the risk of bias assessment of case series using the NIH tool**

| Study ID | Was the study question or objective clearly stated? | Was the study population clearly and fully described, including a case definition? | Were the cases consecutive? | Were the subjects comparable? | Was the intervention clearly described? | Were the outcome measures clearly defined, valid, reliable, and implemented consistently across all study participants? | Was the length of follow-up adequate? | Were the statistical methods well-described? | Were the results well-described? | Quality Rating (Good, Fair, or Poor) |
| --- | --- | --- | --- | --- | --- | --- | --- | --- | --- | --- |
| Ito et al. 2020 | Yes | Yes | Yes | N/A | Yes | Yes | Yes | No | Yes | Fair |
